# Normal pressure hydrocephalus: Diagnostic delay

**DOI:** 10.7705/biomedica.5382

**Published:** 2020-12-09

**Authors:** Alejandra Saldarriaga-Cantillo, Viviana Yepes-Gaviria, Juan Carlos Rivas

**Affiliations:** 1 Escuela de Medicina, Universidad del Valle, Cali, Colombia Universidad del Valle Escuela de Medicina Universidad del Valle Cali Colombia; 2 Departamento de Psiquiatría, Hospital Universitario Psiquiátrico del Valle, Cali, Colombia Departamento de Psiquiatría Hospital Universitario Psiquiátrico del Valle Cali Colombia; 3 Departamento de Psiquiatría, Universidad del Valle, Cali, Colombia Universidad del Valle Departamento de Psiquiatría Universidad del Valle Cali Colombia

**Keywords:** Hydrocephalus/diagnosis, hydrocephalus, normal pressure, spinal puncture, ageism, dementia, primary health care, hidrocefalia/diagnóstico, hidrocéfalo normotenso, punción lumbar, viejismo (sic), demencia, atención primaria de salud

## Abstract

**Introduction:**

Normotensive hydrocephalus is a differential diagnosis in the evaluation of the dementia syndrome. The diagnostic protocols would allow detecting this pathology that has more effective treatment than other dementias.

**Objective:**

To describe a population with clinical suspicion of normal pressure hydrocephalus evaluated in a Colombian psychiatric hospital and discuss the possible reasons for its diagnostic and therapeutic delay.

**Materials and methods:**

We conducted a retrospective study of medical records to identify patients with suspected normal pressure hydrocephalus during a 5-year period.

**Results:**

Thirty-five patients with suspected normal pressure hydrocephalus underwent diagnostic lumbar puncture and five of them were considered candidates for a peritoneal-venous shunt, but none underwent this surgical procedure. After three to six months of the lumbar puncture, the gait pattern improved in 22.8% of the patients, cognition in 22.8%, and sphincter control in 11.4%. Improvement was not sustained in the long term (1 year) in any of them.

**Conclusion:**

This study suggests the poor implementation of the protocols for evaluating patients with cognitive deficits and delays in the diagnosis of normal pressure hydrocephalus. A small number of patients were identified as candidates for treatment. Normal pressure hydrocephalus is a potentially reversible clinical entity with the placement of a peritoneal ventricular shunt, but delays in diagnosis and treatment have deleterious consequences for patients and their families.

## Resumen

**Introducción.:**

La hidrocefalia normotensiva es un diagnóstico diferencial en la evaluación del síndrome demencial. Los protocolos diagnósticos permitirían detectar esta condición, cuyo tratamiento es más efectivo que el de otras demencias.

**Objetivo.:**

Describir una población con sospecha clínica de hidrocefalia normotensiva evaluada en un hospital psiquiátrico colombiano y discutir las posibles razones de la demora en el diagnóstico y en la terapia de esta condición clínica.

**Materiales y métodos.:**

Se hizo un estudio retrospectivo de los registros médicos para detectar pacientes con sospecha de hidrocefalia normotensiva durante un período de cinco años.

**Resultados.:**

A 35 pacientes con sospecha de hidrocefalia normotensiva se les hizo una punción lumbar diagnóstica. Cinco de ellos se consideraron candidatos para una derivación ventrículo-peritoneal, pero ninguno se sometió a este procedimiento quirúrgico. A los 3-6 meses de la punción lumbar, se observó una mejoría del patrón de la marcha en el 22,8 % de los pacientes, de la cognición en el 22,8 % y del control del esfínter en el 11,4 %. La mejoría no se mantuvo a largo plazo (un año) en ningún paciente.

**Conclusión.:**

Se encontró una implementación deficiente de los protocolos de evaluación de los pacientes con déficit cognitivos y demoras en el diagnóstico de la hidrocefalia normotensiva, así como un número reducido de pacientes clasificados como candidatos para el tratamiento. La hidrocefalia normotensiva es una condición clínica potencialmente reversible con la colocación de una derivación ventrículo-peritoneal. Los retrasos en el diagnóstico y en el tratamiento tienen consecuencias perjudiciales para los pacientes y sus familias.

Normal pressure hydrocephalus is a clinical syndrome with the triad of dementia, disordered gait, and urinary incontinence ^1^^,^^2^. Commonly, the disease is accompanied by frontal and subcortical cognitive deficits, which can be confused with other neurological syndromes ^3^^,^^4^. Ventriculomegaly with normal opening pressure on lumbar puncture is an normal pressure hydrocephalus hallmark ^4^. However, the symptoms and radiological findings of this disease may also be present in other common medical entities such as Parkinson's disease, Biswanger's disease, vascular dementia, and even normal aging ^5^^-^^7^.

Normal pressure hydrocephalus incidence varies between 1.36 and 1.58 per 100,000 persons per year ^8^^,^^9^, with an increased incidence in the ninth decade of life ^10^. The discrepancies likely reflect inconsistent definitions of normal pressure hydrocephalus and differences between the study populations ^11^.

Normal pressure hydrocephalus diagnosis is a probability when there are two symptoms of the classic triad associated with ventriculomegaly on cranial computed tomography (CT) or magnetic resonance imaging (MR) suggesting an increase in ventricular size with signs of cerebrospinal fluid (CSF) flow ^12^. The reference standard to determine if a patient with normal pressure hydrocephalus is a candidate for surgery is a lumbar puncture. The clinical improvement of the symptoms following the procedure predicts the benefit derived from the placement of a peritoneal-venous shunt ^13^^,^^14^.

Given that normal pressure hydrocephalus is a dementia syndrome potentially reversible with the placement of a peritoneal-venous shunt, it is important to characterize, recognize, and diagnose it accurately. Current data indicate that peritoneal-venous shunt placement is effective and early treatment can increase survival ^15^. However, there is little consensus on the diagnostic criteria of this disease and the selection of patients who would potentially benefit from a it ^16^.

This study describes a population of patients with suspected normal pressure hydrocephalus evaluated in a psychiatric referral center and the possible reasons for the diagnostic and therapeutic delay.

## Materials and methods

### Population studied

We included a population of patients with clinical suspicion of normal pressure hydrocephalus diagnosed between January 1, 2009, and December 31, 2014, at *Hospital Psiquiátrico Universitario del Valle* in Cali, Colombia. We collected the medical records and conducted a retrospective review. The study was approved by the *Institutional Ethical Review Board* of the *Hospital Psiquiátrico Universitario del Valle* (Act 005-014).

This hospital specializes in the intervention of all aspects of mental health and is the center with the greatest complexity in psychiatric care in southwestern Colombia. The cases were defined as patients who had at least two symptoms of the classic normal pressure hydrocephalus triad, brain imaging evidence of dilation of the ventricular system (Evans index over 0.30), and who had undergone an invasive diagnostic procedure such as lumbar puncture ^17^.

### Lumbar puncture

A high-volume lumbar puncture was performed in the cases included to remove a large volume of CSF (typically 40-50 ml) after one to four hours of gait testing; the test was also performed 24 hours after the procedure. Transient recovery in gait after the lumbar puncture has been considered a positive prognostic indicator for surgery. One to two lumbar puncture attempts were performed and the mean opening pressure of the cerebrospinal fluid was evident in ranges of normal variation (<180 mm H_2_O or 13 mm Hg with the patient in the lateral position).

### Clinical scales

We assessed normal pressure hydrocephalus triad clinical symptoms using the normal pressure hydrocephalus scale (table 1) ^19^. This ordinal scale determines the severity of the patient's clinical picture using scores that independently assess the degree of impairment of gait, sphincter control, and cognition. The scores on the normal pressure hydrocephalus scale range from 3 to 15. The minimum score of 3 corresponds to a patient who does not walk and always stays in bed or in a sitting position with incontinence of the bladder, loss of anal sphincter tone, and minimal awareness. The maximum score of 15 indicates that the patient exhibits normal gait, does not report subjective cognitive alterations, and shows normal control of sphincters.

**Table 1:** Clinical scale for idiopathic normal pressure hydrocephalus ^18^

Symptoms	Score
Gait evaluation	
Patient in bedridden or not able to ambulate	1
Ambulation is possible with help	2
Independent walking is possible but unstable or the patient falls	3
Abnormal but stable gait	4
Normal gait	5
Cognitive function	
Patient is vegetative	1
Severe dementia	2
Important memory problems with more or less severe behavior disturbance	3
Memory problems reported by patient or family	4
Cognitive disturbances are only found by specific tests	5
Sphincter disturbances	
Urinary and faecal incontinence	1
Continous urinary incontinence	2
Sporadic urinary incontinence	5
Urinary urgency	4
No objetive or subjetive sphincter disfuntion	5

Total normal pressure hydrocephalus score: gait evaluation + cognitive function score + sphincter disturbance score

The following data were recorded: sex, age at the time of diagnosis, duration of symptoms, symptoms and severity at the time of diagnosis, response to lumbar puncture, short- and long-term disease course, neuroimaging records, and associated comorbidities. Simple descriptive statistics were calculated using univariate analysis.

## Results

We detected 326 records from the *Hospital Psiquiátrico Universitario del Valle* database under the diagnosis of hydrocephalus. Thirty-five cases (66% female) met the inclusion criteria for normal pressure hydrocephalus, and the average age at the time of evaluation was 77.3 years (range: 47-96 years). The average follow-up time was 33.8 months (range: 3-84 months).

Patients with suspected normal pressure hydrocephalus on admission had a generic diagnosis including senile dementia and Alzheimer's disease. The average duration of symptoms before establishing a diagnostic suspicion of normal pressure hydrocephalus was 66.7 months (range: 0.6-240 months). Thirty to 60% of cases presented with severe symptoms that generated a high burden of dependency on third parties (table 2).

**Table 2:** Characterization of patients with clinical suspicion of idiopathic normal pressure hydrocephalus

Case	Sex	Age (years)	Symptoms duration (months)	Baseline symptoms			Gait outcomes		Cognitive decline		Urinary incontinence	
				Gait disturbance	Cognitive decline	Urinary incontinence	Short term (3-6 months)	Long term (3 years)	Short term (3-6 months)	Long term (3 years)	Short term (3-6 months)	Long term (3 years)
1	M	70	48	1	3	2	1	1	3	1	2	1
2	F	75	24	3	3	1	4	2	4	2	4	1
3	F	73	36	3	3	4	3	3	3	3	4	4
4	M	66	24	4	2	2	2	1	2	1	2	1
5	F	78	108	4	2	1	4	3	2	2	1	1
6	F	62	96	2	3	1	3	2	3	3	3	1
7	F	79	216	3	2	4	4	3	3	1	4	1
8	F	84	1	3	3	4	4	2	4	2	4	4
9	M	74	72	3	3	1	3	2	2	2	1	1
10	M	78	60	1	3	1	2	1	3	3	2	1
11	M	78	72	1	2	1	1	1	2	2	1	1
12	F	83	240	1	2	1	1	1	2	2	1	1
13	F	81	36	1	2	1	1	1	3	2	1	1
14	F	89	72	1	2	1	3	1	3	2	3	1
15	F	76	72	3	2	2	1	1	1	1	2	2
16	M	74	72	3	3	1	2	2	2	2	1	1
17	M	47	240	3	3	3	3	3	3	3	3	1
18	F	82	24	4	2	2	4	4	2	1	2	1
19	M	85	48	4	3	4	4	4	2	2	4	3
20	F	65	84	4	2	3	4	3	2	2	3	3
21	F	87	60	2	2	3	2	1	4	2	3	3
22	F	96	180	2	2	1	2	2	2	2	1	1
23	F	63	36	4	2	1	4	(-)	2	(-)	1	(-)
24	F	84	144	3	2	1	2	2	1	1	1	1
25	F	79	12	3	2	1	3	3	2	2	1	1
26	M	73	24	3	2	2	3	3	2	1	2	2
27	F	81	24	3	2	3	3	0	2	(-)	3	(-)
28	F	82	0,6	4	4	4	4	0	3	(-)	4	(-)
29	M	76	180	3	3	3	4	3	3	3	4	3
30	M	79	12	3	2	1	3	3	2	2	1	1
31	F	79	6	2	2	1	3	(-)	2	(-)	1	(-)
32	F	93	1,44	3	2	3	3	3	2	2	3	3
33	M	80	24	1	2	1	1	(-)	3	(-)	1	(-)
34	F	77	18	3	3	3	3	3	3	3	3	3
35	F	79	1	3	2	3	3	3	3	3	3	3

All the patients had impaired gait, cognitive dysfunction, and loss of sphincter control. Of these, 11 (31%) were immobile, 22 (63%) had severe cognitive impairment, and 22 (63%) sphincter dysfunction that required permanent assistance. All 35 patients underwent CT on admission, four of them also underwent MR, and one, positron emission tomography. In all cases, ventriculomegaly was documented with an Evans index over 0.30. At the hospital admission examination, the Folstein Mini-Mental Scale (MMSE) was recorded in the clinical history of 12 patients with an average score of 19/30 (range). No patients were evaluated with neurocognitive tests.

All 35 patients underwent lumbar puncture. In the short term (3-6 months), 8 (22.8%) of them showed gait improvement, 8 cognitive improvement reported by their caregivers, and 4 (11%) improved sphincter control. Five patients were considered candidates for peritoneal-venous shunt placement due to the overall outcomes after lumbar puncture. However, none of them underwent this procedure: Two were not considered candidates for peritoneal-venous shunt during presurgical evaluation; in another two cases, the patients' guardians did not give consent for the procedure, and in one case, health services did not authorize it.

## Discussion

Our results suggest a poor implementation of protocols for evaluating patients with cognitive deficits, delays in the diagnosis of normal pressure hydrocephalus while a small number of patients were identified as candidates for treatment.

Normal pressure hydrocephalus is a reversible and potentially curable cause of dementia with effective, specific treatment. Early diagnosis can change the patient's overall prognosis and decrease the burden of the disease. The longer normal pressure hydrocephalus goes untreated the worse is its prognosis ^15^.

Normal pressure hydrocephalus represents a diagnostic challenge because it shares symptoms with other neurological syndromes and even with aging itself. Thus, a patient can present with the classic triad of normal pressure hydrocephalus without having the disease. Ventriculomegaly is part of the suspected diagnosis but not when it is the only sign ^20^.

It is striking that the majority of cases evaluated did not have neuroimaging at the time of the first assessment in the *Hospital Psiquiátrico Universitario del Valle* nor was there a diagnostic study using a cognitive deficit protocol to evaluate other differential diagnoses including other reversible dementia syndromes of normal pressure hydrocephalus (B_12_ hypovitaminosis, hypothyroidism, infectious causes, metabolic and toxic causes, etc.) ^1^.

It is possible that a delayed normal pressure hydrocephalus diagnosis (which implies a lower possibility of reversing its symptoms) and the presence of comorbidities are related to the low rates of referral for peritoneal-venous shunt placement ^15^.

Our findings suggest a weak adherence to the diagnostic protocols of patients with cognitive deficits in primary care leading to diagnostic and therapeutic delays in normal pressure hydrocephalus. As the incidence of dementia increases substantially in the aging population, we anticipate that the consequences of late and erroneous diagnosis in dementia will represent a greater burden on public health over time ^21^.

Studies should be conducted to explore whether the lack of compliance with the evaluation protocols of patients with dementia syndromes responds to patients' old age and the perception of these neurological entities as irreversible ^22^ or to the lack of knowledge among primary care physicians of the best diagnostic approach ^18^. Given that patients with early dementia are more likely to benefit from the intervention, future efforts to improve the timeliness of diagnosis should focus specifically on the detection of more subtle and early manifestations of the disease ^23^.

It is estimated that approximately half of the cases of dementia remain undiagnosed ^24^. An important barrier among healthcare providers is the perception that providing an early diagnosis of dementia is more harmful than useful. This attitude responds to the idea that precise diagnosis should be sought only when an inevitable problem has occurred ^18^^,^^24^. Such fear is likely exaggerated, as studies suggest that most patients prefer the full disclosure of dementia diagnosis ^25^. Early diagnosis allows for the optimal use of therapeutic resources and enables due information for individuals and families together with appropriate coping tools and a support network that can alleviate the disabling psychological distress that caregivers may experience ^13^.

A systematic review has shown that the delayed diagnosis of dementia syndromes also responds to the limited resources of the healthcare system (particularly the limited time available for medical consultation, which hampers the detection and management of dementia symptoms). Other barriers include communication problems and poor knowledge of symptoms among patients, healthcare providers, and caregivers ^26^. In the light of the current evidence, it is not acceptable to insist on the diagnosis of senile dementia because this implies denying the patients the possibility of receiving adequate treatment according to the etiology of their ailment.

The positive side of this situation is that it can be improved and, if addressed, early detection of normal pressure hydrocephalus is possible. Educational measures in primary geriatric care regarding normal aging and adherence to the clinical practice guidelines for dementia syndromes may improve timely diagnosis and reduce stigma regarding the perception of irreversibility and therapeutic limitations ^27^^,^^28^.

Among the limitations of our study we should mention the confusion bias implicit in the observational design, but, on the other hand, it was useful to generate hypotheses and plan public health interventions.

Ideally, neuropsychological tests should be done before and after the lumbar puncture; however, health plans in Colombia do not assume this cost, as was evident in all the study cases.

## References

[1] Picascia M, Zangaglia R, Bernini S, Minafra B, Sinforiani E, Pacchetti C (2015). A review of cognitive impairment and differential diagnosis in idiopathic normal pressure hydrocephalus. Funct Neurol.

[2] Adams RD, Fisher CM, Hakim S, Ojemann RG, Sweet WH (1965). Symptomatic occult hydrocephalus with "normal" cerebrospinal-fluid pressure. A treatable syndrome. N Engl J Med.

[3] Iddon JL, Pickard JD, Cross JJ, Griffiths PD, Czosnyka M, Sahakian BJ (1999). Specific patterns of cognitive impairment in patients with idiopathic normal pressure hydrocephalus and Alzheimer's disease: A pilot study. J Neurol Neurosurg Psychiatry.

[4] Barron SA, Jacobs L, Kinkel WR (1976). Changes in size of normal lateral ventricles during aging determined by computerized tomography. Neurology.

[5] Golomb J, Wisoff J, Miller DC, Boksay I, Kluger A, Weiner H (2000). Alzheimer's disease comorbidity in normal pressure hydrocephalus: Prevalence and shunt response. J Neurol Neurosurg Psychiatry.

[6] Espaillat R, Gaviria M (2000). Hidrocefalia de presión normal, una demencia reversible. Revista Colombiana de Psiquiatría.

[7] Sudarsky L, Ronthal M (1983). Gait disorders among elderly patients. A survey study of 50 patients. Arch Neurol.

[8] Pyykko OT, Nerg O, Niskasaari HM, Niskasaari T, Koivisto AM, Hiltunen M (2018). Incidence, comorbidities, and mortality in idiopathic normal pressure hydrocephalus. World Neurosurg.

[9] Lemcke J, Stengel D, Stockhammer F, Guthoff C, Rohde V, Meier U (2016). Nationwide incidence of normal pressure hydrocephalus (NPH) assessed by insurance claim data in Germany. Open Neurol J.

[10] Aragones JM, Altimiras J, Alonso F, Roura P, Alfonso S, Bajo L. (2018). Idiopathic normal pressure hydrocephalus: High incidence in people over 80 years of age. Rev Esp Geriatr Gerontol.

[11] Klassen BT, Ahlskog JE (2011). Normal pressure hydrocephalus: How often does the diagnosis hold water?. Neurology.

[12] Andersson J, Rosell M, Kockum K, Soderstrom L, Laurell K (2017). Challenges in diagnosing normal pressure hydrocephalus: Evaluation of the diagnostic guidelines. eNeurologicalSci.

[13] Thakur SK, Serulle Y, Miskin NP, Rusinek H, Golomb J, George AE (2017). Lumbar puncture test in normal pressure hydrocephalus: Does the volume of CSF removed affect the response to tap?. AJNR Am J Neuroradiol.

[14] Singer OC, Melber J, Hattingen E, Jurcoane A, Keil F, Neumann-Haefelin T (2012). MR volumetric changes after diagnostic CSF removal in normal pressure hydrocephalus. J Neurol.

[15] Jaraj D, Wikkelso C, Rabiei K, Marlow T, Jensen C, Óstling S (2017). Mortality and risk of dementia in normal-pressure hydrocephalus: A population study. Alzheimers Dement.

[16] Krauss JK, Halve B (2004). Normal pressure hydrocephalus: Survey on contemporary diagnostic algorithms and therapeutic decision-making in clinical practice. Acta Neurochir (Wien).

[17] Williams MA, Malm J (2016). Diagnosis and treatment of idiopathic normal pressure hydrocephalus. Continuum (Minneap Minn).

[18] Sahuquillo J, Rubio E, Codina A, Molins A, Guitart JM, Poca MA (1991). Reappraisal of the intracranial pressure and cerebrospinal fluid dynamics in patients with the so-called "normal pressure hydrocephalus" syndrome. Acta Neurochir (Wien).

[19] Palm WM, Saczynski JS, van der Grond J, Sigurdsson S, Kjartansson O, Jonsson PV (2009). Ventricular dilation: Association with gait and cognition. Ann Neurol.

[20] Manuel DG, Garner R, Finés P, Bancej C, Flanagan W, Tu K (2016). Alzheimer's and other dementias in Canada, 2011 to 2031: A microsimulation Population Health Modeling (POHEM) study of projected prevalence, health burden, health services, and caregiving use. Popul Health Metr.

[21] Gove D, Downs M, Vernooij-Dassen M, Small N (2016). Stigma and GPs' perceptions of dementia. Aging Ment Health.

[22] de Vries K, Brooker DJ, Smith P (2013). Dementia skills and competencies for primary care liaison: A model for improving identification and timely diagnosis. Prim Health Care Res Dev.

[23] Brooker D, La Fontaine J, Evans S, Bray J, Saad K (2014). Public health guidance to facilitate timely diagnosis of dementia: Alzheimer's Cooperative Valuation in Europe recommendations. Int J Geriatr Psychiatry.

[24] Coebergh JA (2014). The difficulty and dangers of diagnosing dementia. BMJ.

[25] Wynn MJ, Carpenter BD (2019). Discourse features among providers, patients, and companions and their effect on outcomes of dementia diagnosis disclosure. J Gerontol B Psychol Sci Soc Sci.

[26] Sarkar U, Simchowitz B, Bonacum D, Strull W, López A, Rotteau L (2014). A qualitative analysis of physician perspectives on missed and delayed outpatient diagnosis: The focus on system-related factors. Jt Comm J Qual Patient Saf.

[27] Chithiramohan A, lliffe S, Khattak I (2019). Identifying barriers to diagnosing dementia following incentivisation and policy pressures: General practitioners' perspectives. Dementia.

[28] Bennett CE, De Boos D, Moghaddam NG (2019). Developing a tool to support diagnostic delivery of dementia. Dementia.

